# Redefining Roles: A Paradigm Shift in Tryptophan–Kynurenine Metabolism for Innovative Clinical Applications

**DOI:** 10.3390/ijms252312767

**Published:** 2024-11-27

**Authors:** Masaru Tanaka, Ágnes Szabó, László Vécsei

**Affiliations:** 1 HUN-REN-SZTE Neuroscience Research Group, Hungarian Research Network, University of Szeged (HUN-REN-SZTE), Danube Neuroscience Research Laboratory, Tisza Lajos krt. 113, H-6725 Szeged, Hungary; 2 Department of Neurology, Albert Szent-Györgyi Medical School, University of Szeged, Semmelweis u. 6, H-6725 Szeged, Hungary; szabo.agnes.4@med.u-szeged.hu; 3 Doctoral School of Clinical Medicine, University of Szeged, Korányi fasor 6, H-6720 Szeged, Hungary

**Keywords:** tryptophan, kynurenine, neurodegenerative diseases, oxidative stress, immune response, neuroprotection, antioxidants, microbiota, neuroinflammation, systems biology

## Abstract

The tryptophan–kynurenine (KYN) pathway has long been recognized for its essential role in generating metabolites that influence various physiological processes. Traditionally, these metabolites have been categorized into distinct, often opposing groups, such as pro-oxidant versus antioxidant, excitotoxic/neurotoxic versus neuroprotective. This dichotomous framework has shaped much of the research on conditions like neurodegenerative and neuropsychiatric disorders, as well as cancer, where metabolic imbalances are a key feature. The effects are significantly influenced by various factors, including the concentration of metabolites and the particular cellular milieu in which they are generated. A molecule that acts as neuroprotective at low concentrations may exhibit neurotoxic effects at elevated levels. The oxidative equilibrium of the surrounding environment can alter the function of KYN from an antioxidant to a pro-oxidant. This narrative review offers a comprehensive examination and analysis of the contemporary understanding of KYN metabolites, emphasizing their multifaceted biological functions and their relevance in numerous physiological and pathological processes. This underscores the pressing necessity for a paradigm shift in the comprehension of KYN metabolism. Understanding the context-dependent roles of KYN metabolites is vital for novel therapies in conditions like Alzheimer’s disease, multiple sclerosis, and cancer. Comprehensive pathway modulation, including balancing inflammatory signals and enzyme regulation, offers promising avenues for targeted, effective treatments.

## 1. Introduction

A variety of diseases, including neurodegenerative disorders, autoimmune conditions, cancer, and psychiatric illnesses such as depression and schizophrenia, demonstrate abnormal metabolism in the tryptophan (Trp)-kynurenine (KYN) metabolic pathway [1,2,3,4]. Although this pathway is well acknowledged to be involved in these conditions, it is still unclear whether the observed dysmetabolism is a causal element that contributes to the onset of the disorders or just a result of the progressive development of the illnesses. Clarifying the roles of significant Trp-KYN pathway byproducts and enzymes, such as KYN, quinolinic acid (QUIN), and indoleamine 2,3-dioxygenases (IDOs), has gained increasing attention [5,6,7]. The compounds are under investigation for their possible functions as risk factors for the initiation of diseases, as biomarkers for early detection, or as targets for tentative interventions to slow down the progression [1,8,9,10,11,12]. Investigators are especially intrigued by the impact of these metabolic products on immune responses, neuroinflammation, and cellular energy regulation [2,13,14,15,16,17]. The objective of this review is to acquire more profound understanding of the contributions of the Trp-KYN pathway to different diseases by elucidating its fundamental mechanisms. This understanding may eventually lead to novel strategies for prevention, diagnosis, and treatment, paving the way for more effective interventions in managing complex, multifactorial disorders.

**Figure 1:**
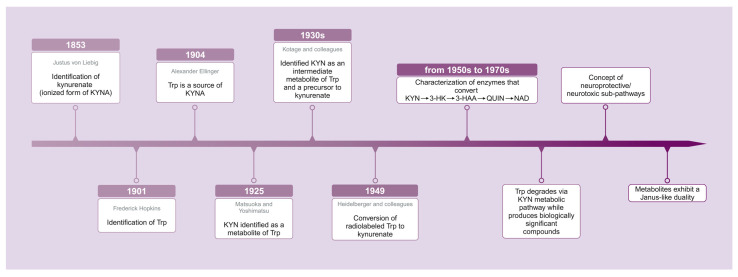
Timeline of tryptophan (Trp)–kynurenine (KYN) metabolic pathway. This timeline highlights key discoveries in the Trp-KYN pathway, beginning with the identification of kynurenate in 1853 by Justus von Liebig, preceding Trp’s isolation by nearly 50 years [18]. In 1901, Frederick Hopkins identified Trp as an essential amino acid [19,20]. In 1904, Alexander Ellinger first identified Trp as a source of kynurenic acid (KYNA) [21]. In 1925, Matsuoka and Yoshimatsu detected KYNA as a Trp metabolite, and in the 1930s [22], Kotake and colleague established KYN as an intermediate and kynurenate precursor [23,24]. Finally, in 1949, Heidelberger and colleagues confirmed the conversion of radiolabeled Trp to kynurenate [25]. The enzymes that convert KYN to nicotinamide adenine dinucleotide (NAD) were characterized by Saito in 1957 [27], Soda in 1979 [28], Long in 1954 [29], and Nishizuka in 1963 [30]. 3-HAA: 3-hydroxyanthranilic acid: KYNA: kynurenic acid; KYN: kynurenine; 3-HK: 3-hydroxykinurenine; NAD: nicotinamide adenine dinucleotide; QUIN: quinolinic acid; Trp: tryptophan.

In the realm of biochemistry, the discovery of key metabolic intermediates often unfolds in unexpected sequences. Notably, kynurenate, the ionized form of kynurenic acid (KYNA) was identified in the urine of dogs as early as 1853 by Justus von Liebig, nearly five decades prior to the isolation of Trp itself [18]. It was British chemist Frederick Hopkins who, in 1901, successfully identified Trp as an essential amino acid integral to protein structure, a breakthrough for which he later received the Nobel Prize [19,20]. Shortly thereafter, in 1904, Alexander Ellinger detected that Trp is a source of KYNA [21]. Notwithstanding these initial findings, the exact biological importance of these compounds, particularly their involvement in metabolic pathways, remained unknown for a considerable period of time. In 1925, Matsuoka and Yoshimatsu identified KYNA as a metabolite of Trp in the urine of rabbits that had been fed a diet rich in Trp [22]. In the 1930s, Kotake and colleague identified KYN as an intermediate metabolite of Trp and a precursor to kynurenate [23,24]. By 1949, Heidelberger and colleagues confirmed the conversion of radiolabeled Trp to kynurenate, solidifying the understanding of this metabolic relationship [25,26]. Subsequently, from the 1950s through the end of the 1970s, enzymes that convert KYN to 3-hydroxykynurenine (3-HK) [27], 3-HK to 3-hydroxyanthranilic acid (3-HAA) [28], 3-HAA to QUIN [29,30], and QUIN to nicotinamide adenine dinucleotide (NAD) [30] began to be characterized [31]. This chronological progression highlights the evolving understanding of the Trp-KYN pathway’s biological significance in human physiology. Further investigation in the mid-20th century revealed that Trp could be enzymatically degraded via the KYN pathway, producing several biologically significant compounds such as KYNA and QUIN (Figure 1).

Nevertheless, the precise biological roles of these metabolites remained uncertain, and they were mostly considered secondary products of regular metabolic processes. Gradually, scientists have started to acknowledge the participation of these metabolites in diverse biological processes, such as the control of the immune system, inflammation of the brain, and cellular energy production [17,32,33,34,35]. Although there is increasing knowledge, there are still profound uncertainties regarding the role of disturbances in the Trp-KYN pathway. Despite being recognized for more than a century, the intricate functions of these molecules in health and disease are still being vigorously studied, providing potentially novel understandings for the management of disorders such as neurodegenerative disorders, cancer, and psychiatric illnesses [36,37,38,39].

Early studies on KYNs have yielded a split interpretation regarding the biological activities of Trp metabolites. Specifically, KYNA is thought to have antioxidant and neuroprotective properties, whereas 3-HK and QUIN act as pro-oxidants and neurotoxins [39,40,41,42,43,44,45]. Furthermore, this divergence has led to the concept of neuroprotective and neurotoxic sub-pathways within KYN metabolism [5,46,47]. Emerging evidence suggests that most KYNs exhibit a Janus-like duality, with opposing biological effects depending on their concentration and the cellular environment in which they operate [48,49,50]. The objective of this narrative review is to recapitulate the historical understanding, which is still prevalent in the research community, while also facilitating a paradigm shift by highlighting the cutting-edge knowledge regarding the biological properties of KYNs. Additionally, this review underscores the necessity of comprehending the molecular actions of KYNs at multiple levels to appreciate their holistic biological roles. Understanding this intricate balance may provide crucial insights into developing therapeutic strategies aimed at modulating KYN pathways for neuroprotection.

## 2. Traditional Paradigm

**Figure 2:**
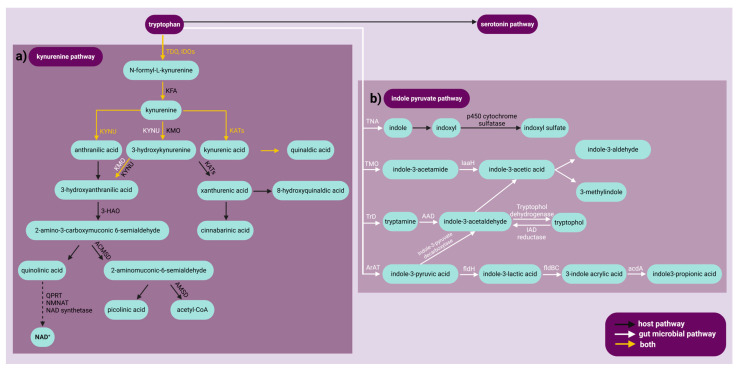
The tryptophan metabolic pathways. Tryptophan (Trp) is catabolized via the serotonin pathway (over 2% of L-Trp), the kynurenine (KYN) pathway (more than 90% of L-Trp), and the gut microbial indole pyruvate pathway (over 5% of L-Trp). (**a**) In the KYN pathway, L-Trp is converted into several key metabolites, including N-formyl-L-kynurenine, KYN, kynurenic acid, anthranilic acid, 3-hydroxykynurenine, xanthurenic acid, 3-hydroxyanthranilic acid, quinolinic acid, picolinic acid, and nicotinamide adenine dinucleotide (NAD^+^). These conversions occur via the action of various enzymes: tryptophan 2,3-dioxygenase (TDO), indoleamine 2,3-dioxygenases (IDOs), kynurenine formamidase (KFA), kynurenine 3-monooxygenase (KMO), kynurenine aminotransferases (KATs), kynureninase (KYNU), 3-hydroxyanthranilate oxidase (3-HAO), quinolinate phosphoribosyl transferase (QPRT), nicotinamide mononucleotide adenylyltransferase (NMNAT), NAD synthetase, amino-β-carboxy-muconate-semialdehyde-decarboxylase (ACMSD), and 2-aminomuconic-6-semialdehyde dehydrogenase (AMSD). KYNA is also further metabolized by the gut microbiome, resulting in quinaldic acid and 8-hydroxyquinaldic acid, which may be dehydroxylated from xanthurenic acid [5,8,62]. Notably, the gut microbiota contributes to the KYN pathway [70]. (**b**) In the gut microbial indole pyruvate pathway, L-Trp metabolism occurs via four distinct pathways: the indoxyl sulfate pathway, the indole-3-acetamide (IAM) pathway, the tryptamine pathway, and the indole-3-propionic acid (IPA) pathway. In the indoxyl sulfate (INS) pathway, the rate-limiting enzyme is tryptophanase (TNA), which requires pyridoxal phosphate. It converts Trp to indole, which passes through the gut lining before being hydroxylated into 3-hydroxyindole (indoxyl), which is then converted to INS in the liver by p450 cytochrome and sulfonation [71]. The IAM pathway starts with tryptophan-2-monooxygenase (TMO), which converts Trp to IAM, which is then converted to indole-3-acetic acid (IAA) by indole-3-acetamide hydrolase (IaaH). IAA can then be metabolized into indole-3-aldehyde or decarboxylated into 3-methylindole (skatole) [72,73]. In the tryptamine pathway, tryptophan decarboxylase (TrD) converts Trp to tryptamine via amino acid decarboxylase (AAD), which is then converted into indole-3-acetaldehyde (IAAld). IAAld can be converted into IAA or reversibly into indole-3-ethanol (tryptophol) [74,75]. Finally, in the IPA pathway, aromatic amino acid aminotransferase (ArAT) converts Trp to indole-3-pyruvic acid, which yields indole-3-lactic acid by phenyllactate dehydrogenase (fldH), 3-indole acrylic acid by phenyllactate dehydratase (fldBC), and, eventually, indole 3-propionic acid by acyl-coenzyme A dehydrogenase (acdA) [75,76,77]. Black arrows: host pathway; white arrows: gut microbial pathway; yellow arrows: both host and microbial pathways; dashed arrow: intermediate metabolites catalyzed by three enzymes.

For many years, researchers have investigated the biological functions of Trp and its metabolites, especially those generated through the KYN pathway. Trp is essential for protein synthesis and serves as a precursor to bioactive compounds such as serotonin, melatonin, and NAD, which are crucial for sustaining physiological functions [51,52,53,54,55,56]. These compounds are essential for maintaining physiological processes. Historically, research has focused primarily on the serotonin pathway due to its importance in regulating mood, sleep, and cognitive functions, particularly in relation to mental health disorders such as depression and anxiety [57,58,59,60,61]. Recent research has concentrated on the KYN pathway, which metabolizes over 90% of Trp not utilized in protein synthesis [5,8,62]. This pathway produces various bioactive molecules that are crucial in immune responses, inflammation, oxidative stress, and neurodegeneration [36,63,64,65]. KYNs are associated with Alzheimer’s disease (AD), Parkinson’s disease (PD), and other neurodegenerative disorders [1,65,66,67,68,69] (Figure 2). Their dual functions—some neuroprotective, some neurotoxic—make the KYN pathway an important area of research for understanding and potentially treating these diseases.

### 2.1. Pro-Oxidants and Antioxidants

KYN metabolites, particularly 3-HK and QUIN, are well documented for their pro-oxidant activities, contributing significantly to oxidative stress [78,79,80]. Historical research has uncovered their ability to generate reactive oxygen species (ROS), implicating them in the progression of neurodegenerative diseases through oxidative damage rather than excitotoxicity [80,81,82] (Table 1).

3-HK is a strong pro-oxidant that produces ROS like superoxide and hydrogen peroxide, which directly damage lipids, proteins, and DNA [92,93,94,95,96,97]. Eastman and Guilarte first described 3-HK in 1989, and it has since been identified as a major contributor to oxidative stress and neurotoxicity in neurodegenerative diseases such as AD, PD, and Huntington’s disease (HD) [67,81,86,93,98,99,100]. 3-HK increases neuronal oxidative damage, disrupts mitochondrial function, and exacerbates the degeneration of brain regions important for memory and motor control [101]. Even at nanomolar concentrations, 3-HK causes significant oxidative damage and accelerates disease progression [102]. 3-HK is now the subject of research into the KYN pathway for therapeutic interventions meant to lessen oxidative stress and neurodegeneration because of its function in promoting ROS-mediated neuronal damage.

Similarly, the pro-oxidant properties of QUIN were identified in 1997 [87,88]. Researchers found that QUIN could increase oxidative stress by promoting the formation of free radicals, particularly in the context of inflammation [89,90,91]. This oxidative activity leads to the damage of mitochondria and other cellular structures, further implicating QUIN in the progression of disorders such as HD [103,104,105]. Studies from the late 1990s and 2000s confirmed that elevated levels of QUIN correlated with increased oxidative damage in neurodegenerative conditions.

KYN metabolites are not only involved in pro-oxidant activities but also exhibit significant antioxidant properties, which play crucial roles in counterbalancing oxidative stress [8,81,106,107]. KYNA has been identified for its neuroprotective effects through antioxidant mechanisms, and historical research has gradually uncovered its contributions to protecting the brain from oxidative damage [41,108,109].

In 1904, Alexander Ellinger identified KYNA as derived from Trp [110]. The antioxidant properties of KYNA were explored in the mid-1990s, with a notable study by Goda and colleagues [84,85]. Further studies opened new avenues for understanding the therapeutic potential of KYN pathway metabolites. Later, Gilles and colleagues demonstrated that KYNA could scavenge free radicals and protect neurons from oxidative stress in vitro [111]. The broad recognition of KYNA as an antioxidant compound likely solidified in the scientific literature around 2005–2010 as more studies began to explore its multifaceted neuroprotective and anti-inflammatory roles [112,113,114,115]. KYNA is particularly noted for its ability to neutralize ROS and reduce lipid peroxidation, which is critical in preventing oxidative damage to cell membranes [40,116,117,118,119]. Studies in the 2000s further confirmed KYNA’s role as an antioxidant, showing that it can mitigate oxidative stress in conditions such as neuroinflammation and neurodegeneration [40,41,120].

These discoveries have opened new avenues for understanding the therapeutic potential of KYN pathway metabolites. In short, these historical findings highlight the diverse antioxidant roles of KYN pathway metabolites. Their ability to scavenge ROS, chelate metal ions, and regulate oxidative processes underscores their significance in protecting the brain from oxidative stress, providing a potential therapeutic avenue for neurodegenerative diseases where oxidative damage is a central pathological feature.

### 2.2. Excitotoxicity and Neuroprotection

The KYN pathway is crucial in Trp metabolism, generating metabolites that play key roles in both the central nervous and immune systems [121,122,123,124]. It contributes to excitotoxicity, a process where excessive activation of receptors leads to neuronal damage, often seen in neurodegenerative diseases like AD and HD [125,126,127,128]. However, the pathway also has neuroprotective functions, helping to regulate excitatory neurotransmission and prevent harmful cellular stress [129,130,131] (Table 1). In addition to its effects on brain health, the KYNs influence immune modulation by interacting with receptors that regulate inflammation and immune responses [122,132,133,134,135]. These multiple roles in excitotoxicity, neuroprotection, and immune regulation make the KYN metabolism a critical focus for research into therapies for neurodegenerative and immune-related disorders, as its balance between toxicity and protection can significantly impact disease progression. Understanding how the KYN pathway functions in different environments is key to harnessing its potential for therapeutic interventions.

#### 2.2.1. Excitotoxicity

The excitotoxic properties of KYN pathway metabolites, especially QUIN, have been widely investigated for their critical involvement in neurodegenerative diseases [136,137,138]. QUIN and other metabolites are produced during the degradation of Trp via the KYN pathway, a process active in both peripheral tissues and the central nervous system (CNS) [80,139,140,141]. Among these metabolites, QUIN stands out as a potent neurotoxin. It exerts its toxic effects primarily through the overactivation of N-methyl-D-aspartate (NMDA) receptors, leading to an excessive influx of calcium ions into neurons [88,89,142,143]. This cascade of events disrupts cellular homeostasis, triggering oxidative stress and the production of free radicals, which further damage cellular components like proteins, lipids, and DNA [88,90,143,144]. Over time, this oxidative damage contributes to the dysfunction and death of neurons, a hallmark of neurodegenerative conditions such as AD, HD, and multiple sclerosis (MS) [145,146,147]. Understanding the precise mechanisms of KYN pathway-mediated neurotoxicity is critical for developing strategies to mitigate its contribution to neuronal degeneration and excitotoxicity.

QUIN, a metabolite of the KYN pathway, was first identified in the 1970s as a potent neurotoxin due to its excitotoxic properties [148,149,150]. Further research uncovered QUIN’s ability to overactivate NMDA receptors, a subtype of glutamate receptor that plays a critical role in synaptic plasticity and memory formation [151,152,153]. By the late 1980s, researchers began exploring QUIN’s broader impact on brain cells beyond neurons, particularly its effects on glial cells like astrocytes and microglia [154,155,156]. Glial cells, which are essential for maintaining neuronal function and homeostasis, were found to be susceptible to QUIN-induced toxicity as well. This discovery expanded QUIN’s role from a purely excitotoxic agent to a molecule contributing to neuroinflammation and broader brain degeneration. In addition to overactivating NMDA receptors, QUIN was shown to interfere with mitochondrial function, impairing the cell’s energy production mechanisms and amplifying the generation of ROS [103,157,158]. This oxidative stress exacerbates cellular damage, affecting both neurons and glial cells. Moreover, the inflammatory response triggered by QUIN in the glial cells further perpetuates neurodegeneration [44,82,159]. These findings spurred increased interest in QUIN’s role in neurodegenerative disorders, leading to more targeted investigations into its potential as a therapeutic target for diseases like PD, amyotrophic lateral sclerosis (ALS), and even certain forms of epilepsy [160,161,162].

#### 2.2.2. Neuroprotection

KYN metabolites have garnered significant attention in neurobiological particularly through their modulation of NMDA receptors. Among the KYN pathway metabolites, KYNA stands out due to its potent antioxidant properties and non-competitive antagonism of NMDA receptors. The KYN pathway, responsible for the metabolism of Trp into several neuroactive compounds, plays a dual role in neuroprotection and neurotoxicity, with KYNA contributing to the preservation of neuronal health (Table 1). By the late 1980s, KYNA functioned as a natural antagonist of the NMDA receptor [163]. This finding was particularly significant because NMDA receptors play a key role in synaptic plasticity and cognitive functions [152,164,165,166]. The discovery opened new research avenues focused on enhancing KYNA’s therapeutic potential, either by modulating its levels or developing synthetic analogs for use in clinical interventions.

In research in the early 1990s, researchers further emphasized the neuroprotective effects of KYNA. Expanding on their previous work, they demonstrated that KYNA’s inhibition of NMDA receptors was instrumental in preventing neurological damage in various experimental models of brain disorders, such as AD and HD [167,168,169,170,171,172]. At the same time, comparisons between KYNA and QUIN began to surface. While KYNA functioned as an NMDA receptor antagonist, offering protection against neurological damage, QUIN acted in the opposite manner as an NMDA receptor agonist, promoting harmful processes. This duality between KYNA and QUIN highlighted the complexity of the KYN pathway and its significant influence on brain health. KYNA’s protective role contrasted sharply with QUIN’s neurodegenerative effects, underscoring how modulation of these metabolites could have profound implications for brain function and the progression of neurological diseases. These discoveries paved the way for further exploration of the KYN pathway as a therapeutic target, inspiring research into how manipulating KYNA and QUIN levels could be used to mitigate the effects of neurological disorders.

The development of the concept that the KYN pathway divides into neurotoxic and neuroprotective branches arose from early studies on the duality between KYN metabolites: KYNA as neuroprotective, and 3-HK and QUIN as neurotoxic [173,174,175]. Researchers emphasized that these two contrasting properties of KYN metabolite had opposing effects on NMDA receptors, with KYNA functioning as an antagonist and QUIN as an agonist. This finding led to the idea that the KYN pathway could have both protective and damaging roles in the brain, depending on which metabolite predominated. KYNA, by blocking NMDA receptors, was shown to protect neurons, while QUIN’s receptor activation was associated with processes that contribute to neurodegeneration [176,177,178,179,180,181]. This dual action of the KYN metabolites not only helped explain its complex role in neurological diseases but also provided a framework for understanding how imbalances in these metabolites could drive brain disorders. For example, elevated levels of QUIN and reduced levels of KYNA have been linked to conditions like AD and HD, where excitatory signaling contributes to pathology. This understanding of the pathway’s dual nature has since driven research aimed at developing therapeutic strategies to shift the balance toward neuroprotection by modulating KYNA and QUIN levels.

## 3. Emerging Evidence

Emerging evidence challenges the longstanding binary categorizations of KYN metabolites, presenting new perspectives on their multifaceted roles. Recent studies reveal that these metabolites, such as KYN and KYNA, do not operate strictly as pro-oxidants or antioxidants, but rather exhibit dual properties depending on factors like concentration and cellular environment. Additionally, neuroimaging studies, such as those utilizing voxel-based morphometry, have advanced our understanding of structural brain alterations in various disorders, highlighting the importance of examining gray matter volume and concentration in conditions like autism spectrum disorder [182]. This complexity underscores the need to reconsider traditional views on how these compounds influence neuroprotection, excitotoxicity, and immune regulation. The evolving understanding highlights a dynamic and context-dependent behavior of KYN pathway metabolites, which could revolutionize therapeutic approaches [183]. Researchers are now focusing on concentration gradients and metabolic environments to explore novel applications for disease management, particularly in neurodegenerative and immune-mediated disorders [124,184,185,186,187]. These developments mark a significant shift in the conceptual framework governing KYN metabolism [188].

### 3.1. Pro-Oxidants or Antioxidants?

The KYN pathway produces several metabolites that exhibit dual properties, acting as either pro-oxidants or antioxidants depending on their concentration and the specific cellular environment. These contrasting roles have been highlighted in numerous studies conducted after 2000, revealing that the oxidative or reductive effects of KYN metabolites can vary dramatically based on factors such as cell type, redox state, and the presence of other signaling molecules. Understanding these dual properties is critical for appreciating how KYN dysmetabolism contributes to neurodegenerative diseases, inflammation, and immune responses [189].

3-HAA is the first metabolite in the KYN pathway, known for its Janus-like duality as both a pro-oxidant and an antioxidant, which has garnered significant attention in recent research. Studies in the mid-1990s demonstrated the pro-oxidant properties of 3-HAA, showing its ability to generate ROS, leading to oxidative stress and contributing to neurotoxicity in certain conditions [190]. Subsequently, research expanded on 3-HAA’s complex role by revealing its function as a co-antioxidant [191]. 3-HAA was shown to stabilize antioxidant systems and mitigate oxidative damage more effectively. This co-antioxidant activity highlighted the compound’s nuanced role in modulating oxidative stress, depending on cellular conditions and its interactions with other antioxidant molecules. Furthermore, Esaki and colleagues highlighted its antioxidant properties, showing that 3-HAA can scavenge free radicals and inhibit lipid peroxidation, protecting cells from oxidative damage [192,193]. This versatility positions 3-HAA as an important factor in physiological processes such as immune regulation and neuroprotection [80,194,195]. Its dual antioxidant and pro-oxidant properties, which shift depending on the cellular context, suggest a complex role in neurodegenerative diseases and immune modulation. These findings, which are supported by several studies, suggest that 3-HAA may be important in balancing oxidative stress, with potential therapeutic applications for conditions such as AD and MS.

KYN itself exhibits dual behavior as a pro-oxidant or antioxidant, largely depending on its concentration and the cellular environment [116,196,197,198]. At physiological levels, KYN can act as an antioxidant, protecting neurons from oxidative stress by scavenging free radicals. However, at higher concentrations, KYN can shift towards a pro-oxidant role, contributing to oxidative damage in neurons and glial cells. This dual role is especially important in neurodegenerative diseases, where elevated KYN levels are linked to increased oxidative stress and neuronal death (Table 2).

KYNA has been widely studied for its neuroprotective antioxidant properties, but recent research indicates it can also act as a pro-oxidant under certain conditions. At low concentrations, KYNA effectively reduces oxidative stress in cultured neurons by neutralizing ROS [40]. This antioxidant function is beneficial in protecting neurons from damage in conditions such as AD. However, at high concentrations, KYNA can paradoxically enhance oxidative stress by disrupting mitochondrial function and promoting the production of free radicals [199]. This concentration-dependent duality is influenced by the local cellular redox environment, particularly in tissues already undergoing oxidative stress. KYNA also functions as an agonist at the orphan G-protein-coupled receptor GPR35 [201,202,203].

Anthranilic acid (AA) exhibits both antioxidant and pro-oxidant properties, depending on environmental conditions. AA can exhibit pro-oxidant properties under specific conditions, particularly in the presence of strong reductants or iron(II) ions [111,254]. In such environments, it participates in redox reactions that generate ROS, exacerbating oxidative stress and contributing to cellular damage. Conversely, AA also demonstrates antioxidant activity, primarily through its ability to chelate metal ions like Cu(II) and iron [255]. This chelation inhibits Fenton-like reactions, preventing the formation of hydroxyl radicals and reducing oxidative stress [254]. By scavenging ROS and protecting cellular components, AA functions as a secondary antioxidant, mitigating oxidative damage. This dual functionality is context-dependent, with redox activity influenced by environmental factors. AA’s capacity to both promote and counteract oxidative stress highlights its complex role in redox biology and oxidative processes.

3-HK has been identified as one of the most potent pro-oxidants within the KYN pathway. Studies after 2000 showed that at elevated concentrations, 3-HK acts as a strong pro-oxidant, promoting lipid peroxidation and oxidative stress, particularly in neurons [204]. This effect is exacerbated in neurodegenerative diseases like PD, where increased 3-HK levels contribute to oxidative damage and cell death. However, at very low concentrations, 3-HK can exhibit mild antioxidant properties by modulating redox signaling pathways [193,256]. 

Xanthurenic acid (XA) has demonstrated both pro-oxidant and antioxidant properties depending on its concentration and cellular conditions. A study showed that XA at low concentrations can act as an antioxidant, supporting mitochondrial function by reducing oxidative damage and maintaining ATP production in neurons [193,208,209,210]. However, when XA accumulates at higher levels, it shifts towards a pro-oxidant role, potentially disrupting mitochondrial respiration and leading to increased production of ROS [78,206,207]. The delicate balance determines whether XA will protect or damage cells, with implications for metabolic disorders and neurodegenerative diseases. 

Cinnabarinic acid (CA) is a less-studied metabolite, but recent research has begun to uncover its dual redox properties [195,257]. A study found that CA can act as an antioxidant in specific controlled environments, particularly in immune cells, where it reduces oxidative stress and supports cellular redox balance [215,216,258,259]. However, under conditions of chronic inflammation or when present at high concentrations, CA can shift towards a pro-oxidant role, promoting oxidative damage [214,260]. This dual behavior suggests that CA’s redox properties are highly context-dependent, influenced by the immune status and overall redox environment of the tissue.

Picolinic acid (PA) has antioxidant and pro-oxidant properties that vary depending on the context [261]. PA enhances the generation of ROS and high-valent metal species, thereby accelerating oxidation reactions and pollutant degradation [217,218,219]. In growing-finishing pigs, high chromium picolinate (CrPic) intake did not increase oxidative damage markers, but altered antioxidant enzyme activity, hinting at a pro-oxidant effect [220]. In diabetic rats, however, CrPic reduced oxidative stress and enhanced antioxidant enzyme activity, indicating antioxidant properties [221]. In neurodegenerative diseases, PA plays a dual role within the KYN pathway, potentially acting as both an antioxidant and a pro-oxidant, as seen in AD, PD, and ALS [261]. This variability highlights the complexities of PA’s function in various biological systems.

The dual pro-oxidant and antioxidant properties of KYN pathway metabolites such as KYN, KYNA, AA, 3-HK, XA, CA, 3-HAA, and PA highlight the complexity of their roles in cellular processes. Recent findings emphasize the importance of concentration and cellular environment in determining whether these metabolites protect against or contribute to oxidative stress. These insights have significant implications for understanding the role of the KYN pathway in diseases such as neurodegeneration, cancer, and inflammation, where the balance between antioxidant and pro-oxidant effects can influence disease progression.

### 3.2. Receptor Agonists or Antagonists?

The KYN pathway metabolites have emerged as critical regulators of cellular processes, exhibiting dual properties as receptor agonists or antagonists depending on their concentration and the cellular environment. These metabolites, such as KYN, KYNA, AA, XA, and CA can modulate signaling pathways through their interactions with receptors like NMDA, alpha-amino-3-hydroxy-5-methyl-4-isoxazolepropionic acid (AMPA), mGluRs, and/or aryl hydrocarbon receptor (AhR). Notably, research conducted after 2000 has further illuminated these dual roles. 

KYN itself plays a significant role in modulating receptor activity in the brain, influencing both excitatory and immune pathways. KYN is an agonist for the AhR, a transcription factor involved in immune regulation and neuroinflammation. By binding to AhR, KYN can modulate gene expression related to inflammatory responses, linking it to immune-related processes in the CNS [262,263,264]. In contrast, the molecular docking technique revealed that KYNA does not directly activate the AhR; rather, KYN mediates this activation, implying a revised model for KYN pathway interactions with AhR [265]. These interactions highlight KYN’s role in balancing excitatory neurotransmission and immune responses, contributing to its involvement in both neuroprotective and neuroinflammatory processes.

KYNA acts as a significant modulator in the brain, primarily through its antagonist activities at several key receptors. It is a potent antagonist at NMDA receptors, particularly at the glycine co-agonist site, reducing excitotoxicity linked to excessive glutamate signaling, which is implicated in neurodegenerative diseases [266,267,268]. It was reported, however, that at low concentrations, KYNA acts as an agonist at NMDA receptors [49]. KYNA also antagonizes AMPA receptors, further damping excitatory neurotransmission [269]. However, KYNA’s dual actions were also reported regarding AMPA receptors [270]. Additionally, KYNA has inhibitory effects on metabotropic glutamate receptors (mGluRs), particularly the mGlu2 and mGlu3 subtypes, which are involved in regulating synaptic plasticity and neurotransmitter release [271,272,273]. Through these interactions, KYNA plays a neuroprotective role by limiting overactive excitatory signaling. Beyond its effects on glutamate receptors, KYNA is an endogenous AhR agonist [274]. Activation of AhR by KYNA links it to anti-inflammatory processes, contributing to its broader neuroprotective properties [275,276,277].

XA is an intriguing metabolite in the KYN pathway, known for its interactions with several key receptors. XA has also been shown to interact with mGluRs, particularly mGluRs type 2 and mGluRs type 3, where it may act as a modulator of synaptic transmission and plasticity [211,212,213]. Through these interactions, XA can regulate glutamate-related excitatory signaling and contribute to neuroprotective effects. XA is also linked to AhR, where it may act as an agonist, influencing immune and inflammatory pathways in the brain [278,279,280]. These combined receptor interactions make XA an important regulator of both neural and immune functions. 3-HK has been identified as an agonist at AhR, which is involved in the regulation of gene expression and immune responses [281,282,283]. These interactions highlight the complex role of 3-HK in neurophysiological processes and its potential impact on neurological health. CA interacts with mGluRs. Specifically, it acts as an agonist at mGlu4 receptors [284]. This interaction can modulate neurotransmission and has potential implications for neuroprotective strategies. CA acts as an agonist at AhR, which plays a role in regulating immune responses and gene expression [285].

The versatile properties of KYN pathway metabolites as receptor agonists or antagonists, depending on concentration and cellular context, reflect their intricate roles in modulating neurochemical and immune signaling. Emerging studies have significantly contributed to our understanding of how metabolites like KYN, KYNA, AA, XA, and CA interact with receptors such as NMDA, AMPA, mGluRs, and/or AhR. These findings emphasize the importance of concentration-dependent effects in determining whether these metabolites act protectively or contribute to pathology. Further research into these dynamics offers exciting potential for therapeutic strategies that manipulate receptor activity to address conditions such as neurodegeneration, inflammation, and immune dysfunction.

### 3.3. Immunomodulators

The KYN pathway has emerged as a critical metabolic route for Trp degradation, producing metabolites with significant effects on immune regulation. Over the decades, it has become clear that certain KYN metabolites, such as KYN, KYNA, AA, XA, and CA function as ligands for the AhR, a key transcription factor involved in immune regulation [286,287,288]. The discovery of their interaction with AhR marked a turning point in understanding the broad physiological roles of the KYN pathway, offering new insights into immune system modulation.

Duarte and colleagues reported that KYN acts as a potent ligand for AhR, influencing immune regulation through its effects on T-cell differentiation [289]. By activating AhR, KYN promotes the development of regulatory T cells (Tregs) and dampens the activity of pro-inflammatory Th17 cells, a process that helps maintain immune tolerance. This balance is crucial in preventing excessive immune responses, which can lead to autoimmune disorders. Elevated KYN levels have been associated with various diseases, including cancer, where it contributes to an immunosuppressive microenvironment that allows tumor cells to evade immune detection. 

This research highlighted the importance of KYN-AhR interactions in both physiological and pathological immune processes. KYNA also plays a role in immune modulation. KYNA’s ability to bind AhR influences immune responses by reducing the production of inflammatory cytokines and regulating dendritic cell and macrophage activity. By promoting a tolerogenic environment, KYNA can help control inflammation and immune activation. This discovery helped shift the view of KYNA beyond its neuroactive properties to include its role in immune regulation. KYNA’s interaction with AhR continues to be an area of interest, particularly in chronic inflammatory diseases and conditions requiring immune suppression.

Early research into their immune-modulating properties suggested that AA and XA may influence cytokine production and immune cell function, contributing to the regulation of immune responses [290,291]. Although the detailed mechanisms are still under investigation, the potential of AA and XA to interact with AhR positions them as promising targets for further research into immune modulation, particularly in autoimmune and inflammatory conditions. CA has been shown to serve as a unique ligand for AhR. CA has the ability to modulate immune tolerance by promoting the differentiation of Tregs and limiting pro-inflammatory immune responses. This interaction with AhR suggests its potential use in therapeutic strategies for autoimmune diseases such as MS and rheumatoid arthritis. By fostering immune tolerance, CA may help suppress pathological immune activation, making it a valuable target for future immune therapies.

Beyond the primary metabolites like KYN and KYNA, other derivatives of the KYN pathway also interact with AhR to influence immune functions. For example, 3-HK and 3-HAA have been investigated for their roles in immune regulation [194,292]. These metabolites participate in a complex regulatory network, influencing both pro-inflammatory and anti-inflammatory responses, depending on the immune context. This further underscores the importance of the KYN-AhR axis in maintaining immune balance and preventing diseases marked by immune dysregulation, such as chronic inflammation and autoimmune disorders. The discovery of KYN pathway metabolites as ligands for the AhR in the early 2010s significantly broadened our understanding of their roles in immune modulation. This KYN-AhR axis offers exciting therapeutic possibilities, particularly for immune-related diseases such as autoimmunity and chronic inflammation, making it a promising area for future research into immune modulation therapies.

### 3.4. The Body–Brain Axes

**Figure 3:**
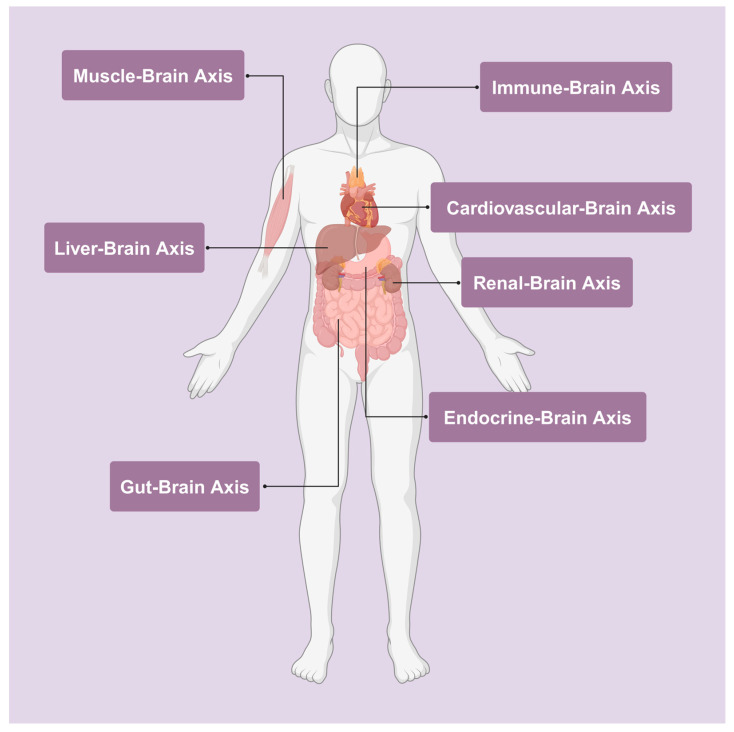
Integration of body-brain axes via the kynurenine (KYN) pathway. The KYN pathway is an important integrative hub that connects peripheral systems to the central nervous system via multiple body-brain axes. These include the gut-brain axis, where gut microbes control metabolites of KYN that affect mood and neuroinflammation; the muscle-brain axis, where kynurenine aminotransferases (KATs) upregulated by exercise transform neurotoxic KYN into neuroprotective kynurenic acid; the cardiovascular-brain axis, where inflammation and vascular health modulate the neuroactive effects of KYN; the renal-brain axis, where kidney function influences the clearance of KYN and systemic inflammation; the endocrine-brain axis, where hormonal regulation affects the production of KYN; and the liver-brain and immune-brain axes, where immunological responses and hepatic metabolism influence neuroinflammatory outcomes. These axes collectively highlight the part the KYN pathway plays in the etiology of mental health conditions, neurodegenerative diseases, and possible treatment approaches.

In recent years, the KYN pathway, the primary route for Trp metabolism, has been recognized as a crucial mediator of several body–brain axes, including the gut microbiome–brain axis, the muscle–brain axis, and other systemic connections [293,294,295,296,297]. These axes are essential for understanding how peripheral metabolic and immune processes influence brain function. The gut microbiome–brain axis, in particular, has received significant attention that gut microbes can regulate the production of KYN and its metabolites, influencing neuroinflammation and mood disorders like depression [298,299]. Meanwhile, the muscle–brain axis highlights the role of physical activity in modulating KYN levels. It was discovered that exercise-induced upregulation of enzymes in skeletal muscle, such as kynurenine aminotransferases (KATs), converts neurotoxic KYN into KYNA, a neuroprotective metabolite, thereby reducing stress-induced depression [300]. Other important axes include the liver–brain and immune–brain axes, where peripheral inflammation and hepatic metabolism influence the KYN pathway, modulating neuroinflammation and cognitive function [301,302]. Collectively, these body–brain axes underline the KYN pathway’s role as a key integrative link between peripheral systems and the CNS, offering insights into the development of neurodegenerative diseases, mental health disorders, and potential therapeutic interventions (Figure 3).

#### 3.4.1. The Gut–Brain Axis

The gut microbiome–brain axis, a bidirectional communication network between the gut microbiota and the CNS, has emerged as a key regulator of brain function and behavior [303,304,305]. In recent years, researchers have uncovered the critical role of the KYN pathway in mediating this axis, particularly in the context of mental health and neuroinflammation [306,307,308]. Gut microbiota can influence Trp metabolism, diverting it through the KYN pathway and producing metabolites that affect brain processes. This connection between the gut and brain opens new avenues for understanding psychiatric and neurodegenerative diseases, highlighting how gut health impacts mental and neurological well-being [309,310] (Figure 3).

A breakthrough in understanding the microbiome’s role in Trp metabolism is that the gut microbiota directly influences the KYN pathway by modulating the availability of Trp, the pathway’s precursor [311,312]. It was shown that gut bacteria can either increase or decrease the flow of Trp into the KYN pathway, depending on the composition of the microbiota. For example, a diverse and healthy microbiome can prevent the excessive diversion of Trp into neurotoxic KYN metabolites, while dysbiosis (an imbalanced gut microbiota) can increase KYN production, leading to heightened neuroinflammation [2,313,314]. This modulation of Trp metabolism by gut bacteria highlights how changes in the microbiome can directly influence brain health, especially in conditions like depression, anxiety, and neurodegenerative diseases [315,316,317].

The gut microbiota has been discovered to influence mental health by regulating KYN metabolism. Elevated levels of KYN and its neurotoxic metabolite, QUIN, are linked to depression, while gut bacteria that convert KYN into KYNA show antidepressant effects [293,318]. These findings suggest therapeutic potential in targeting gut microbiota to balance KYN metabolites and treat psychiatric disorders, and further support the importance of the gut microbiome in neurological health, exploring the role of dysbiosis, or gut microbial imbalance, in metabolic diseases [319]. It was demonstrated that gut dysbiosis can lead to increased production of KYN and QUIN, metabolites known to contribute to neuronal damage and neuroinflammation [320]. These findings suggest that this imbalance may exacerbate conditions like AD and PD. Moreover, studies have explored probiotics as a way to positively modulate the gut–brain axis and KYN metabolism [321]. 

This review highlights the therapeutic potential of probiotics in treating mental and neurological disorders through microbiota-targeted interventions. In the gut microbial indole pyruvate pathway, L-Trp metabolism occurs via four distinct pathways: the indoxyl sulfate (INS) pathway, the indole-3-acetamide (IAM) pathway, the tryptamine pathway, and the indole-3-propionic acid (IPA) pathway [307,322,323,324,325] (Figure 2). Each pathway produces metabolites with unique biological activities. 

##### The Indoxyl Sulfate Pathway

In the INS pathway, tryptophanase (TNA) converts Trp to indole, which is then metabolized in the liver into indoxyl and subsequently INS. INS exhibits a dual role as both a pro-oxidant and antioxidant, with effects that are context- and concentration-dependent [222]. In chronic kidney disease (CKD), INS primarily acts as a pro-oxidant, promoting oxidative stress and inflammation and contributing to cardiovascular issues [222,223,224]. However, under normal physiological conditions, INS can have antioxidant effects by scavenging radicals and protecting against lipid oxidation [225]. This dual behavior underscores the complex role of INS, which shifts based on biological context and pathological conditions. INS is a potent endogenous agonist of the AhR, activating target genes like cytochromes P450 1A1 and 1B1 and contributing to oxidative stress, inflammation, and endothelial dysfunction [226,227,228]. AhR-mediated signaling plays an important role in CKD complications such as cardiovascular disease, bone disorders, and thrombosis, making AhR antagonists a promising therapeutic strategy [326,327,328] (Table 2).

##### The Indole-3-Acetamide Pathway

The IAM pathway starts with tryptophan-2-monooxygenase (TMO), which converts Trp to IAM, which is then converted to indole-3-acetic acid (IAA) by indole-3-acetamide hydrolase (IaaH). IAA can be further metabolized into indole-3-aldehyde (IAld) or 3-methylindole (skatole) [329,330,331] (Figure 2). IAM has shown significant antioxidant activity in various studies, effectively neutralizing free radicals and inhibiting lipid peroxidation [229,230]. Some derivatives also activate heme oxygenase, providing additional protection against oxidative stress [231]. Studies consistently support the antioxidant role of IAM, with no evidence suggesting pro-oxidant behavior. IAA exhibits both antioxidant and pro-oxidant properties, with its effects depending on specific biological conditions. While IAA and its derivatives have strong antioxidant activity, scavenging radicals, and inhibiting lipid peroxidation, certain conditions can cause pro-oxidant effects, such as in the bleomycin–Fe system [230,233]. This dual behavior underscores the context-dependent impact of IAA on oxidative stress. According to current research, IAld is neither an antioxidant nor a pro-oxidant. Further research is needed to determine its role in oxidative stress and whether it has significant antioxidant or pro-oxidant effects. 3-Methylindole (skatole) displays both pro-oxidant and antioxidant properties depending on the biological context. In bacterial biofilms, it exhibits pro-oxidant effects by increasing oxidative stress, suppressing catalase activity, and decreasing biofilm formation in *E. coli* O157 [234]. In hepatocytes, skatole acts as an antioxidant, reducing oxidative stress and protecting against lipotoxicity-induced damage [235]. IAM is a potent AhR agonist, whereas IAld is both a low-potency agonist and a ligand-specific antagonist [232]. Skatole is a medium-efficacy AhR agonist and partial agonist that modulates AhR activity; however, the role of IAA in AhR activity is unclear [232,236] (Table 2).

##### The Tryptamine Pathway

Within the tryptamine pathway, tryptophan decarboxylase (TrD) catalyzes the amino acid decarboxylase (AAD) reaction that converts Trp to tryptamine. Tryptamine is subsequently transformed into indole-3-acetaldehyde (IAAld), which can either be further converted to IAA or reversibly to indole-3-ethanol (tryptophol) [74,75,322,332,333] (Figure 2). Tryptamine, a naturally occurring alkaloid, shows both pro-oxidant and antioxidant activities, with its effects varying depending on the biological context. Tryptamine has pro-oxidant effects in certain systems, such as cyanobacteria, where it causes oxidative stress and lipid peroxidation [237]. However, as an antioxidant, it effectively scavenges hydroxyl and hydroperoxyl radicals, sometimes surpassing standard antioxidants like ascorbic acid and Trolox in efficacy [238,239,240], highlighting the context-dependent nature of its biological impact. The role of IAAld as an antioxidant or pro-oxidant is unclear, and its behavior in redox biology has not been studied as extensively as other indole derivatives. Tryptophol has antioxidant and radical scavenging properties, effectively neutralizing the ABTS•+ radical at physiological pH [245]. Its antioxidant potential, as measured by Trolox equivalent antioxidant capacity (TEAC), suggests that tryptophol and similar indoles are effective radical scavengers, outperforming standard antioxidants such as Trolox and ascorbic acid [233]. Tryptamine and tryptophol are AhR agonists with weak to medium efficacy, inducing AhR-mediated gene expression; however, tryptamine also has partial antagonist activity [232,241,242,243]. IAAld, while not a strong direct agonist, plays an important role in the metabolic pathways that lead to the formation of potent AhR activators [232,241,244], emphasizing the selective and context-dependent modulation of AhR by Trp metabolites (Table 2).

##### The Indole-3-Propionic Acid Pathway

In the IPA pathway, aromatic amino acid aminotransferase (ArAT) converts Trp to indole-3-pyruvic acid (IPyA), yielding indole-3-lactic acid (ILA), 3-indole acrylic acid (IAcA), and, eventually, IPA (Figure 2). Relatively little is understood about the redox properties of IPyA in biological environments, particularly regarding how it interacts with ROS and influences oxidative stress pathways under various physiological or pathological conditions. Research findings suggest that ILA possesses antioxidant and anti-inflammatory properties [248], indicating its potential role in scavenging ROS and reducing oxidative damage, as well as modulating inflammatory responses by downregulating pro-inflammatory cytokines and related signaling pathways. IAcA demonstrates antioxidant properties that support intestinal health by promoting epithelial barrier function and reducing inflammation [249]. Certain mucin-utilizing commensal bacteria, such as *Peptostreptococcus russellii*, produce IAcA, which helps protect against epithelial injury and mitigates inflammatory responses, suggesting its therapeutic potential for conditions like inflammatory bowel disease. IPA has high antioxidant activity, protecting cells from oxidative damage caused by amyloid beta, iron overload, and hydrogen peroxide [250,251,252]. It has demonstrated neuroprotective and anti-inflammatory effects, preventing lipid peroxidation, reducing DNA damage, and inhibiting proinflammatory cytokine synthesis [334,335]. IPA does not display pro-oxidant behavior, making it a promising therapeutic candidate for oxidative stress-related diseases. IPyA is a potent AhR agonist with anti-inflammatory effects [246,247], while IPA serves as a partial agonist, enhancing macrophage function and protecting against sepsis [253]. There is no evidence suggesting that ILA or IAcA act as AhR agonists or antagonists (Table 2).

The gut microbiome–brain axis plays a crucial role in regulating KYN pathway activity, influencing neuroinflammation, mental health, and neurodegeneration. Findings from researchers have shown that the composition of the gut microbiota can dramatically alter the balance of KYN metabolites, with profound effects on brain function. These discoveries highlight the potential for microbiota-targeted therapies, such as probiotics, to modulate the KYN pathway and offer new treatments for psychiatric and neurodegenerative disorders. Understanding this axis opens new avenues for exploring how gut health directly impacts the brain, positioning the microbiome as a key player in mental and neurological well-being.

#### 3.4.2. The Muscle–Brain Axis

The muscle–brain axis has emerged as a significant area of research, highlighting how skeletal muscles influence brain function, particularly through the modulation of the KYN pathway [336,337,338]. Physical activity has been shown to regulate the production and metabolism of KYN, altering the balance between neurotoxic and neuroprotective metabolites [339,340,341]. This connection offers important insights into how exercise impacts mental health, neuroinflammation, and neurodegenerative diseases [337]. Studies have revealed the molecular mechanisms linking muscle activity to brain health, emphasizing the role of muscle-derived enzymes in shaping KYN metabolism and reducing the harmful effects of stress and neurotoxicity [16,342,343,344] (Figure 3).

It was found that physical exercise promotes KATs in skeletal muscle, reducing neurotoxic KYN levels in the brain, which protects against stress-induced behavioral changes and depression [16,342,343]. This highlights the muscle–brain axis as a key pathway for exercise’s antidepressant effects. By detoxifying KYN, exercise promotes mental health and stress resilience. Recent research has focused on how physical exercise alters KYN metabolism via the muscle–brain axis. One study revealed that skeletal muscle increases the expression of KATs [345]. This prevents KYN from crossing the blood–brain barrier (BBB) and causing neuroinflammation. The protective effects of exercise on mental health have been highlighted, with studies showing how muscle-driven changes in KYN metabolism alleviate stress-induced depression and offer promising therapeutic avenues for neuropsychiatric disorders.

The muscle–brain axis plays a critical role in regulating brain health through the modulation of KYN metabolism. Research has highlighted how physical activity can influence KYN metabolism, reducing stress, neuroinflammation, and the risk of neurodegenerative diseases. These findings underscore the importance of exercise in maintaining mental and neurological health, with the muscle–brain axis serving as a key pathway linking physical activity to brain protection. As the understanding of this axis deepens, it offers promising therapeutic strategies for treating mental health disorders and neurodegenerative diseases through targeted modulation of the KYN pathway.

#### 3.4.3. Other Axes

Beyond the gut microbiome–brain and muscle–brain axes, several other systemic connections influence brain function. These include the immune–brain axis, liver–brain axis, cardiovascular–brain axis, renal–brain axis, and endocrine–brain axis, where disruptions in the KYN pathway can lead to altered neuroinflammatory responses and neurodegeneration [346,347,348,349]. Since 2000, research has increasingly focused on how these peripheral systems affect the KYN pathway’s metabolite balance. Understanding these systemic pathways sheds light on how conditions such as immune dysfunction, liver disease, and cardiovascular disorders contribute to brain health and disease through the KYN pathway (Figure 3).

A pivotal paper was published on the role of the KYN pathway in neuroinflammation and neurodegenerative diseases, highlighting its impact on the immune–brain axis [350]. The immunomodulatory properties of KYN metabolites in psychiatric disorders have been explored [351]. Another research focused on the pathway’s influence on inflammatory diseases and its potential therapeutic targets [352]. Rowan Kearns examined the connection between gut permeability, inflammation, and neuroinflammation through the KYN pathway [120]. These studies collectively advance our understanding of KYN metabolism in the immune–brain axis.

The liver–brain axis is another critical pathway that regulates KYN metabolism, with liver dysfunction profoundly impacting brain health (Figure 3). A study explored how liver diseases, including hepatic encephalopathy and cirrhosis, affect KYN pathway activity. It was found that Bisphenol F (BPF) exposure triggered depression-like behaviors in mice through tests like the sucrose preference and forced swim test. Mechanistically, BPF disturbed the KYN metabolic pathway along the liver–brain axis, significantly increasing neurotoxic metabolites such as KYN 3-HK in the brain. These changes were linked to elevated hepatic enzymes, IDO1, and tryptophan 2,3-dioxygenase (TDO)2, and the upregulation of LAT1, facilitating the transport of KYN across the BBB [347]. The findings suggest targeting LAT1 or liver-derived KYN may be a potential therapeutic approach for BPF-induced depression. These findings show that impaired liver function disrupts this balance, leading to psychiatric outcomes, including cognitive decline and increased susceptibility to depression.

The cardiovascular–brain axis, particularly through the BBB, plays a vital role in regulating the transfer of KYN metabolites between the periphery and the CNS (Figure 3). This axis plays a crucial role in various physiological processes, including nutrient delivery, oxygenation, and waste removal. It was discussed how cardiovascular diseases such as hypertension and atherosclerosis disrupt BBB integrity [353]. Reduced BBB function facilitates the influx of inflammatory cytokines and neurotoxic metabolites from the blood, increasing neuroinflammation and cognitive impairment. This research also indicated that cardiovascular health is crucial for maintaining KYN pathway homeostasis, suggesting that therapeutic strategies to improve vascular integrity could help control the neurotoxic effects of KYN metabolism in neurological diseases.

CKD significantly alters systemic KYN metabolism, which can negatively impact brain function [354,355,356,357] (Figure 3). Pawlak and colleagues investigated the accumulation of toxic degradation products of KYN in hemodialyzed patients [358]. The patients undergoing hemodialysis exhibited elevated levels of KYN pathway metabolites, which are associated with oxidative stress and inflammation. This accumulation may contribute to the pathophysiology of cardiovascular and neurological complications commonly observed in these patients. This study underscores the critical role of KYN metabolism in linking renal and neurological health.

The endocrine–brain axis, particularly through the regulation of stress hormones and insulin, affects KYN metabolism and brain function [359,360] (Figure 3). Depression was investigated in relation to the KYN pathway, demonstrating that immune activation, particularly via cytokines, causes an increase in KYN and QUIN production, which influences serotonin and glutamate neurotransmission. This immune activation is linked to the pathophysiology of major depression. Furthermore, the lipopolysaccharide (LPS)-induced activation of the KYN pathway was investigated, specifically how immune system activation affects KYN metabolism in the brain [295]. Their research, conducted in mice after LPS administration, provided valuable insights into how immune responses can influence neurochemical processes in brain regions that regulate mood. This work highlights the potential connection between inflammation and psychiatric disorders, such as depression, by demonstrating how immune-induced changes in neurochemistry may contribute to mood dysregulation. These studies suggest that targeting the endocrine regulation of the KYN pathway could help alleviate the neurotoxic effects of chronic stress and metabolic dysfunction on the brain.

The systemic connections between the immune, liver, cardiovascular, renal, and endocrine systems and the brain play crucial roles in regulating KYN metabolism, influencing neuroinflammation, cognitive function, and neurodegeneration. Research on the body–brain axis has shed light on how disruptions in these peripheral systems lead to altered KYN pathway activity, contributing to various neurodegenerative and neuroinflammatory conditions [166,361,362,363,364,365]. These findings highlight the importance of maintaining systemic health to regulate the KYN pathway and prevent its neurotoxic effects, offering potential therapeutic approaches for neurodegenerative diseases and cognitive disorders.

## 4. Paradigm Shift

To apply the concepts of first-order, second-order, third-order, and higher-order responses to understand the levels of interaction in biological systems, a clear framework is essential [366,367,368]. Molecule–molecule interactions, such as enzyme–substrate binding, often follow first-order kinetics, where the rate is directly proportional to the concentration of molecules involved [369,370,371]. In neural transmission, more complex second-order responses occur, involving neurotransmitter-receptor interactions that affect downstream signaling pathways [372,373,374]. The immune system showcases higher-order interactions, where cytokines and immune cells create feedback loops, modulating immune responses [375,376,377]. These varying orders of interaction form the basis of systems biology, which seeks to map how molecules, cells, and tissues work together to maintain homeostasis or trigger responses to stimuli [378,379,380]. Furthermore, the concept of zero-order responses, particularly in metabolic resilience, highlights how biological systems can maintain stability despite external perturbations, providing insights into disease mechanisms and potential therapeutic targets [381,382,383,384].

### 4.1. Molecule–Molecule Interactions

Molecule–molecule interactions play a fundamental role in biological systems, governing processes such as enzyme activity, signal transduction, and gene expression. The study of these interactions dates back to the early 20th century, with key contributions from scientists like Emil Fischer, who introduced the “lock and key” model of enzyme specificity in 1894 [385,386]. This concept has since evolved, with modern techniques allowing us to explore more complex molecular interactions, such as allosteric regulation and cooperative binding [387,388,389]. Understanding these interactions is essential for probing the metabolic pathways involved in disease pathogenesis, particularly in disorders linked to Trp-KYN metabolism, which is associated with immune regulation and neurodegeneration.

First-order molecular interactions are characterized by a direct, proportional relationship between interacting molecules. In biological systems, receptor–ligand binding at low concentrations follows first-order kinetics, where the binding rate depends linearly on ligand concentration [390,391,392]. For example, drug binding to a cellular receptor often follows this model at initial stages [393]. Similarly, enzyme–substrate interactions, when substrate concentration is low, can be modeled as first-order processes [394]. In the context of Trp metabolism, first-order interactions are important when analyzing early steps of the KYN pathway, where enzymes like TDO catalyze the conversion of Trp to KYN. Studying these dynamics helps identify points of intervention for potential therapeutic targets in immune-related diseases.

More complex molecular interactions often follow second-order dynamics, where multiple molecules influence each other’s behavior. Cooperative binding, such as the oxygen binding to hemoglobin, exemplifies second-order kinetics, as the binding of one oxygen molecule increases the affinity for subsequent molecules [395,396,397]. In the Trp-KYN pathway, second-order dynamics can be observed in inhibition and allosteric regulation. For example, KYN and its metabolites can act as inhibitors, modulating the activity of key enzymes involved in immune responses. The regulatory effect of such molecules on enzymes like IDO suggests new avenues for modulating this pathway in conditions such as cancer and neuroinflammation.

As molecular interactions become increasingly complex, third-order or higher-order behavior arises. Signal transduction cascades, where a ligand binds to a receptor and activates a series of downstream molecules, provide a prime example of third-order systems. In the KYN pathway, complex interactions between multiple enzymes and signaling molecules influence immune and neurological responses. Protein complexes involved in feedback loops, such as those regulating the balance of KYN and its downstream neuroactive products, exhibit non-linear dynamics. Understanding these interactions is critical for unraveling the multifaceted role of the KYN pathway in pathogenesis, especially in disorders like major depressive disorder and neurodegeneration.

Systems biology offers a holistic approach to studying molecule–molecule interactions, integrating network models and quantitative analyses [398,399,400]. In the context of Trp-KYN metabolism, systems biology can map the complex regulatory networks and feedback loops between enzymes and metabolites. Network models of the KYN pathway can reveal how perturbations at one node, such as elevated levels of KYN, affect downstream processes like neurotoxicity or immunomodulation. Quantitative modeling of enzyme kinetics, receptor–ligand interactions, and feedback mechanisms provides insights into how therapeutic interventions could modulate these pathways, offering new targets for treating conditions such as autoimmune disorders and neurodegenerative diseases [401,402,403,404].

### 4.2. Molecule–Neural Transmission Interactions

Molecule–neural transmission interactions have been a focus of scientific research for over a century. Early work by Sir Charles Sherrington in the late 19th century laid the foundation for understanding how neurons communicate through synapses [405,406,407]. The discovery of neurotransmitters like acetylcholine by Otto Loewi in 1921 further illuminated how chemical signals transmit across neural junctions [408,409,410]. Today, this knowledge is critical for exploring complex metabolic pathways, such as the Trp-KYN pathway, which plays a significant role in immune and neurological functions. Understanding the molecular interactions in this pathway could provide insights into the pathogenesis of neurodegenerative diseases and identify therapeutic targets.

In simple neural transmission scenarios, neurotransmitter release and receptor binding can be modeled as first-order interactions, especially when neurotransmitter concentration is low [411,412]. For instance, when a neurotransmitter like glutamate is released from the presynaptic neuron, it binds directly to postsynaptic receptors, following first-order kinetics [413,414]. This is crucial for understanding early events in Trp metabolism, where low concentrations of KYN may interact with neural receptors in a direct manner. Investigating these first-order interactions can help in identifying how early-stage disruptions in the KYN pathway might lead to altered neural communication and disease development.

As molecular interactions become more complex, second-order dynamics emerge. Neuromodulation, where neurotransmitters such as dopamine or serotonin activate receptors that modulate ion channels or enzymes, follows second-order kinetics [415,416,417]. Similarly, synaptic plasticity—long-term potentiation or long-term depression—involves second-order interactions where repeated stimulation changes synapse strength [418,419,420]. These processes are important in the KYN pathway, where the accumulation of metabolites like QUIN can modulate receptor function and impact synaptic plasticity. Understanding these second-order processes is key to exploring how the KYN pathway contributes to neuroinflammation and neurodegeneration.

Third-order interactions involve complex neural networks where signals propagate through multiple neurons and feedback loops [421,422]. For instance, neurotransmitter cascades triggered by dopamine involve G-protein-coupled receptors, second messengers (e.g., cAMP), and the activation of kinases or transcription factors that regulate gene expression [423,424]. In the Trp-KYN pathway, complex feedback between metabolites and neurotransmitters could influence multiple neural circuits, contributing to memory formation, decision-making, and potentially neurotoxic outcomes. Understanding these interactions helps elucidate how metabolic dysregulation in the KYN pathway might lead to cognitive impairment or neuropsychiatric conditions.

Systems biology offers a framework to model the complexity of neural transmission interactions [425,426]. Mathematical models, such as the Hodgkin–Huxley equations, simulate how neural signals propagate in response to various inputs [427,428,429]. In the context of Trp-KYN metabolism, systems biology approaches can model how disruptions in KYN levels affect broader neural circuits. Functional connectivity analyses and neural network models allow researchers to predict how KYN pathway alterations could lead to neurological diseases like AD or PD. By simulating these interactions, researchers can explore potential therapeutic targets to modulate the pathway and mitigate disease progression.

### 4.3. Molecule–Immune System Interactions

The study of molecular interactions within the immune system has evolved significantly over the last century. The discovery of the antigen–antibody reaction by Emil von Behring in the 1890s laid the foundation for understanding immune defense mechanisms [430,431,432]. This knowledge expanded with advancements in molecular immunology, which identified key pathways like cytokine signaling and T-cell activation [433,434,435]. Today, the immune system’s molecular interactions are being investigated to understand how metabolic pathways, such as the Trp-KYN pathway, contribute to immune regulation and disease pathogenesis. These insights can help identify therapeutic targets for conditions like autoimmune disorders, cancer, and neurodegenerative diseases.

Simple immune responses, such as the binding between an antigen and its corresponding antibody, often follow first-order kinetics [436,437,438]. In these cases, the rate of response is directly proportional to the concentration of the antigen, as seen in early stages of immune recognition [439]. The binding of antibodies to microbial antigens is a classic example of first-order interaction, playing a crucial role in neutralizing pathogens [440,441]. In the context of Trp-KYN metabolism, first-order kinetics might help explain how early immune responses influence downstream effects, such as the production of KYN metabolites that modulate immune activity and inflammation.

Immune signaling pathways often involve second-order interactions, where multiple components interact to trigger a response [442,443]. Cytokines, such as interleukin-2 or tumor necrosis factor-α, bind to receptors on immune cells, initiating signaling cascades that lead to cellular activation. This ligand–receptor interaction is second-order because it involves two interacting molecules [340]. Similarly, T-cell activation, where antigen-presenting cells engage with T-cell receptors through multiple co-stimulatory signals, follows second-order dynamics [444,445,446]. In Trp metabolism, cytokines can influence the activity of enzymes like IDO, thus linking immune activation with metabolic regulation, particularly in cancer and chronic inflammation.

Complex immune processes, such as inflammatory responses, are examples of third-order or higher interactions [447,448,449]. In cytokine storms, a cascade of immune signals involves multiple cell types and feedback mechanisms, creating a highly regulated and amplified response [450,451,452]. This third-order dynamic is critical in understanding immune regulation and pathologies like sepsis or autoimmune flare-ups. Similarly, immune memory and tolerance involve higher-order processes where feedback loops adjust the immune system’s response over time [453,454]. In the KYN pathway, such complex regulation may determine the balance between immune activation and suppression, making it a potential target for therapeutic intervention in immune-mediated diseases.

Systems biology provides a framework for modeling the complex interactions in the immune system. Using computational models, scientists can simulate how molecules such as cytokines, antibodies, and immune cells interact within feedback loops [455,456,457]. This approach is valuable for understanding conditions like autoimmune diseases, where dysregulation in molecular signaling leads to aberrant immune responses. In Trp-KYN metabolism, agent-based models can simulate how immune cells, influenced by KYN metabolites, respond to inflammatory cues. These models help predict disease progression and identify potential targets for therapeutic intervention in conditions such as neuroinflammation and cancer.

### 4.4. Connecting to Systems Biology

Systems biology is a multidisciplinary approach that integrates molecular, cellular, and physiological interactions into comprehensive models to predict biological behaviors at the organismal level [379,458]. Pioneered by researchers like Hiroaki Kitano in the early 2000s, systems biology has revolutionized how we study complex systems by utilizing computational tools and mathematical modeling [459,460]. In the context of Trp-KYN metabolism, systems biology can provide insights into how metabolites like KYN influence immune responses, neuroinflammation, and neurodegeneration. By modeling interactions between neurons, immune cells, and metabolites, systems biology can help identify key regulatory nodes, potentially revealing new therapeutic targets for diseases like AD and cancer.

Mathematical modeling is central to systems biology [461,462,463]. First-order interactions, such as enzyme–substrate reactions, can be modeled using simple linear differential equations [464]. For more complex interactions, like those involving cytokines or neurotransmitters, non-linear models are required to capture the dynamics of second- and third-order responses [465,466,467]. In the Trp-KYN pathway, modeling can be used to simulate how different enzyme activities, like IDO, affect KYN production and downstream immune modulation. These models help predict how alterations in the pathway might contribute to pathogenesis and immune escape in diseases like cancer.

Pathway analysis allows researchers to map complex signaling networks, such as the mitogen-activated protein kinase (MAPK) or Janus kinase/signal transducers and activators of transcription (JAK/STAT) pathways, which often involve multiple orders of molecular interactions [468]. By studying how perturbations, such as drug treatments or mutations, affect these pathways, systems biologists can simulate potential therapeutic outcomes [469,470,471]. In Trp-KYN metabolism, this approach can be used to explore how metabolites interact with immune cells, influencing signaling cascades involved in inflammation and immune suppression. This analysis helps in identifying potential intervention points to modulate the pathway in diseases such as chronic inflammation and autoimmune disorders.

Biological systems are regulated by feedback loops, which can be either negative or positive [379,472,473]. In higher-order interactions, these loops ensure system stability or drive processes like immune regulation and homeostasis [474,475,476]. Systems biology applies control theory to understand how feedback mechanisms influence biological responses [477,478,479]. For instance, in the KYN pathway, feedback loops between KYN metabolites and immune cells can either amplify or dampen immune activity, depending on the context. Understanding these dynamics is essential for identifying therapeutic targets, as manipulating feedback loops could restore balance in conditions like neuroinflammation.

One of the most powerful aspects of systems biology is its ability to integrate multi-omics data, including genomics, proteomics, and metabolomics [480,481,482]. This data-driven approach allows scientists to connect molecular interactions across different scales, from genes to metabolites, and see how they contribute to whole-body physiological responses. In the case of Trp-KYN metabolism, integrating omics data can help uncover how genetic mutations or altered protein expression influence the production of KYN metabolites and their role in pathogenesis. This holistic view is critical for identifying novel drug targets and personalizing therapeutic strategies.

### 4.5. Zero-Order Responses, Resilience Measurement, and Intolerance, Among Others

Zero-order kinetics were first classically applied in pharmacokinetics in the mid-20th century to describe drug metabolism under conditions of enzyme saturation [483,484,485]. Zero-order kinetics occur when the rate of response remains constant, irrespective of changes in the concentration of the stimulus. This saturation happens when all available receptors or enzymes are fully occupied, and the system can no longer increase its response. Even as the concentration of a drug or stimulus rises, the response stays constant. This concept was first identified in pharmacokinetics to describe drug metabolism behavior. Historically, zero-order kinetics have been essential in explaining how certain drugs are metabolized, offering insights into systems that maintain a stable output despite varying external conditions.

A classical example of zero-order kinetics is seen in alcohol metabolism by the liver [486]. When alcohol is consumed, the liver metabolizes it at a constant rate, regardless of the amount consumed, due to the saturation of alcohol dehydrogenase enzymes. Similarly, enzyme reactions exhibit zero-order kinetics when enzyme activity reaches its maximum velocity (Vmax). In such cases, increasing the substrate concentration does not accelerate the reaction. This is particularly relevant in metabolic pathways like the Trp-KYN metabolism, where enzyme saturation may occur, leading to stable output levels even under varied conditions, such as during immune responses or inflammation.

Zero-order responses are critical for measuring metabolic system resilience, particularly in maintaining stability despite external fluctuations [487,488]. In the Trp-KYN pathway, enzymes like IDO can operate under zero-order kinetics when substrate concentrations are high. This allows the system to maintain a steady output of KYN and related metabolites, even under conditions of nutrient depletion or oxygen variability. Such resilience is vital for understanding how this pathway remains functional during immune challenges or stress, offering promising targets for therapeutic interventions in diseases like cancer and neurodegenerative disorders, where metabolic regulation plays a crucial role in pathogenesis.

## 5. Discussion

In recent years, research into the Trp-KYN pathway has expanded our understanding of its critical role in various biological processes [4,34,489]. This metabolic route, which handles over 90% of dietary Trp not used for protein synthesis, produces bioactive metabolites that have significant implications in immune responses, inflammation, oxidative stress, and neurodegeneration [6,490,491]. Historically, the focus was on serotonin production due to its impact on mood and cognitive function, but attention has shifted towards the KYN pathway due to its association with conditions like AD and PD [492,493,494]. KYN metabolites exhibit dual behavior: some, like KYNA, are neuroprotective and act as antioxidants, while others, such as 3-HK and QUIN, promote oxidative stress and neurotoxicity [40,44,261]. The recent paradigm shift has opened up new therapeutic avenues beyond focusing on the balance of pro-oxidant and antioxidant actions of KYN metabolites. Fine-tuning the Trp-KYN pathway may provide novel treatments for neurodegenerative and immune-related diseases, potentially changing our clinical approach to these conditions [8,45,495].

Emerging evidence has significantly broadened the scope of research into Trp-KYN metabolism, shifting traditional views on its metabolites. Historically viewed with distinct pro-oxidant or antioxidant roles, KYN metabolites are now recognized for their dual, context-dependent behaviors [116,496,497]. These behaviors depend largely on concentration gradients and cellular environments [49,119,270]. For instance, KYN and KYNA can act as antioxidants at physiological concentrations, protecting neurons from oxidative stress. However, at elevated levels, these same metabolites can switch to pro-oxidant roles, contributing to oxidative damage, particularly in neurodegenerative diseases like PD and AD. This complexity in KYN metabolism challenges prior binary categorizations and highlights the need for a more nuanced understanding of how these metabolites influence neuroprotection, immune regulation, and oxidative stress. By exploring the concentration-dependent effects of these metabolites, researchers are uncovering new opportunities for therapeutic intervention, particularly for conditions driven by inflammation and oxidative stress. This evolving perspective on KYN metabolism emphasizes its dynamic role in both disease progression and potential treatment avenues.

This review highlights the importance of Trp metabolism, particularly within the context of neurodegenerative diseases and immune dysfunction. Given the widespread availability of L-Trp supplements, it is crucial to consider whether individuals with these conditions should be advised to avoid supplementation due to potential alterations in the KYN pathway [498,499,500]. Patients’ baseline and post-L-Trp loading KYN metabolite profiles may help guide personalized recommendations and offer important insights into the metabolic changes that take place. More investigations into the effects of L-Trp loading on the dynamics of the animal KYN pathway may also help us better understand the related physiological and biochemical alterations. Furthermore, KYNA’s neuroprotective and cytoprotective properties make it a promising nutraceutical that may promote cognitive health by scavenging neurotoxic metabolites [501]. Investigating correlations with microbiome composition may reveal how gut microbiota interacts with Trp metabolism, potentially influencing disease progression or therapeutic outcomes [502,503,504]. Thus, paying closer attention to these aspects of Trp metabolism could enhance our ability to develop targeted strategies for managing neurodegenerative and immune-related conditions.

The paradigm shift in Trp-KYN metabolism underscores a transformative understanding of its metabolites. Once viewed through the rigid lens of pro-oxidant versus antioxidant properties, emerging research highlights the dynamic and context-dependent behavior of KYN metabolites. Those metabolites, previously recognized primarily for their protective roles in neurodegeneration, now reveal dual functions. At physiological levels, these metabolites act as antioxidants, safeguarding neurons from oxidative stress. However, at elevated concentrations, such as in conditions of chronic inflammation or neurodegenerative diseases, KYN metabolites switch roles, exacerbating oxidative damage. This redefinition challenges the traditional binary framework, emphasizing the need for deeper exploration of concentration gradients and cellular environments in understanding KYN metabolism. This shift also opens new therapeutic possibilities by modulating KYN metabolites, offering innovative interventions for diseases where oxidative stress and inflammation are key drivers. Ultimately, this evolving perspective not only reshapes our understanding of neuroprotection and neurotoxicity but also sets the stage for novel clinical applications targeting metabolic imbalances.

Future research into Trp-KYN metabolism should prioritize bridging the gap between in vitro and in vivo preclinical studies by adopting more integrative and dynamic models [505,506,507,508]. Advanced in vivo technologies, such as brain-on-a-chip systems, offer promising tools to replicate the complex metabolic and neural interactions under physiologically relevant conditions [509,510,511]. These platforms will enhance our understanding of how KYN metabolites like 3-HK influence neuroinflammation and oxidative stress within neural circuits. Moreover, refining animal studies by incorporating biomarker-driven stratification, akin to clinical trials, will improve the reliability and translatability of findings to human therapies [512,513,514]. This approach will also enable more targeted interventions, where treatments are personalized based on metabolic profiles. Focusing on conditions like AD and PD, where Trp-KYN dysregulation plays a critical role, future studies can identify predictive biomarkers, facilitating the development of personalized therapeutic strategies aimed at mitigating oxidative damage and neurotoxicity for improved clinical outcomes.

Ongoing clinical trials focusing on the Trp-KYN pathway demonstrate significant efforts to develop therapeutic agents for cancer and immune-related diseases. IDO1 inhibitors such as epacadostat have been tested in advanced stages as standalone treatments or in combination with immune checkpoint inhibitors, but some studies, including Phase III trials, have struggled to meet primary endpoints [515,516,517]. Nevertheless, research continues, focusing on combination strategies. BMS-986205 is another IDO1 inhibitor that has been tested in Phase II/III trials for solid tumors, typically in combination with anti-PD-1 antibodies [518,519,520]. M4112, a dual IDO1/TDO2 inhibitor, has shown promise in Phase I trials to improve immune responses by inhibiting Trp metabolism, which can impair T-cell function [515,521,522]. Furthermore, KNS366, a selective KMO inhibitor developed by Kynos Therapeutics, has completed Phase I trials aimed at controlling inflammation by lowering pro-inflammatory metabolites [515,523,524]. These trials underscore the promise of Trp-KYN pathway modulation in addressing complex diseases, including cancer, neuroinflammation, and systemic immune disorders.

The expanding knowledge of Trp-KYN metabolism paves the way for innovative clinical applications that extend beyond focusing on individual metabolite properties. This complex pathway plays a significant role in modulating immune responses, neuroinflammation, and neuroprotection, making it a promising target for integrated therapeutic strategies. Personalized treatment plans that use metabolic profiling to customize interventions according to patient-specific KYN pathway activity are examples of potential clinical applications. For example, this profiling could guide decisions about L-Trp supplementation, ensuring safe and effective use for individuals with neurodegenerative or immune-related conditions. Furthermore, comprehensive pathway modulation—rather than just adjusting KYNA or QUIN levels—may entail balancing pro-inflammatory and anti-inflammatory signals or fine-tuning enzyme activities like IDO or KATs. These approaches could improve outcomes by restoring homeostasis in neurodegenerative diseases like AD and PD or in autoimmune disorders. Advances in biomarker identification and targeted therapies are critical in translating metabolic insights into practical clinical applications, ultimately improving diagnostic precision and therapeutic efficacy.

The ultimate goal of this line of research is to bridge the gap between understanding Trp-KYN metabolism and applying this knowledge to clinical interventions for neurodegenerative and immune-related diseases. The main challenge lies in reconciling discrepancies between in vitro and in vivo studies, particularly due to the complex interplay of metabolic and neural processes that are difficult to replicate outside a living organism [47,200,525]. To address this, advanced knowledge in systems biology and cutting-edge technologies such as brain-on-a-chip models will be essential. This field has made significant strides, but further research is necessary to clarify the precise roles of KYN metabolites. This review builds on previous work by refining methodologies and introducing new perspectives on receptor interactions and oxidative stress. Its implications for future research are vast, particularly in identifying biomarkers and therapeutic targets. While the review highlights key advancements, it also acknowledges limitations in current experimental models, with promising potential for clinical applications in neurodegenerative and psychiatric disorders.

## 6. Conclusions

This review provides an in-depth exploration of the complex interactions within the Trp-KYN pathway and highlights the importance of revising traditional views on its metabolites. The authors emphasize the necessity of moving beyond the binary categorization of KYN metabolites, such as pro-oxidant/antioxidant or neuroprotective/neurotoxic. They argue for a paradigm shift that recognizes the nuanced, context-dependent roles of these metabolites in various physiological and pathological processes. This perspective has profound theoretical implications, especially in understanding neurodegenerative diseases and immune dysregulation. The review also identifies potential areas for future research, such as examining the concentration-dependent effects of metabolites and how these interactions could be leveraged for therapeutic interventions. Methodologically, further development is needed to explore the interplay between KYN metabolites and other molecular systems in disease contexts. Expanding this understanding will provide new opportunities for disease intervention, particularly in conditions where metabolic dysregulation is central to pathophysiology.

## Figures and Tables

**Table 1:** Traditional view on tryptophan–kynurenine metabolites.

Metabolites	Pro-Oxidant	Antioxidant	Receptors		Ref.
			Agonist	Antagonist	
Kynurenic acid (KYNA)	-	+	-	+ ^1, 2^	[40,83,84,85]
3-Hydroxykynurenine (3-HK)	+	-	-	-	[86]
Quinolinic acid (QUIN)	+	-	+ ^1^	-	[87,88,89,90,91]

NMDA: N-methyl-D-aspartate, +: positive effect, -: unknown, ^1^: NMDA receptor, ^2^: α7 nicotinic acetylcholine receptors.

**Table 2:** Emerging evidence of tryptophan–kynurenine metabolites.

Metabolites	Pro- Oxidant	Anti- Oxidant	Receptors		AhR Agonist	Ref.
			Agonist	Antagonist		
Kynurenine (KYN)	+	+	-	-	+	[116,196,197,198]
Kynurenic acid (KYNA)	+	+	+ ^1^, + ^2^	+ ^3^, ? ^4^	+	[40,83,199,200,201,202,203]
Anthranilic acid (AA)	+	+	-	-	+	[190,191,192,193]
3-Hydroxykynurenine (3-HK)	+	+	-	-	-	[67,193,204,205]
Xanthurenic acid (XA)	+	+	+ ^5^	-	+	[78,193,206,207,208,209,210,211,212,213]
Cinnabarinic acid (CA)	+	+	+ ^6^	-	+	[214,215,216]
3-Hydroxyanthranilic acid (3-HAA)	+	+	-	-	-	[193,205]
Quinolinic acid (QUIN)	+	-	+	-	-	[87,88,89,90,91]
Picolinic acid (PA)	+	+	-	-	-	[217,218,219,220,221]
Indoxyl sulfate (INS)	+	+	-	-	+	[222,223,224,225,226,227,228]
Indole-3-acetamide (IAM)	-	+	-	-	+	[229,230,231,232]
Indole-3-acetic acid (IAA)	+	+	-	-	-	[230,233]
Indole-3-acetaldehyde (IAld)	-	-	-	-	+ ^7^	[232]
3-methylindole (skatole)	+	+	-	-	+	[232,234,235,236]
Tryptamine	+	+	-	-	+ ^8^	[237,238,239,240,241,242,243]
Indole-3-acetaldehyde (IAAld)	-	-	-	-	+	[232,241,244]
Indole-3-ethanol (tryptophol)	-	+	-	-	+	[232,233,245]
Indole-3-pyruvic acid (IPyA)	-	-	-	-	+	[246,247]
Indole-3-lactic acid (ILA)	-	+	-	-	-	[248]
3-indole acrylic acid (IAcA)	-	+	-	-	-	[249]
Indole-3-propionic acid (IPA)	-	+	-	-	+	[250,251,252,253]

NMDA: N-methyl-D-aspartate, AhR: Aryl hydrocarbon receptor +: positive effect, -: unknown, ?: not confirmed in vivo; emerging findings in bold, ^1^: Alpha-amino-3-hydroxy-5-methyl-4-isoxazolepropionic acid (AMPA) receptor, ^2^: G-protein coupled receptor 35 (GPR35) ^3^: NMDA receptor, ^4^: questionable in vivo, ^5^: metabotropic glutamate receptors (mGluRs) types 2 and 3, ^6^: mGlu4R type 4, ^7^: low-potency agonist and ligand-specific antagonist, ^8^: agonist and partial antagonist.

## Data Availability

Data sharing is not applicable to this article.

## Abbreviations

AA	anthranilic acid
AAD	amino acid decarboxylase
AD	Alzheimer’s disease
AhR	aryl hydrocarbon receptor
AMPA	alpha-amino-3-hydroxy-5-methyl-4-isoxazolepropionic acid
ALS	amyotrophic lateral sclerosis
ArAT	aromatic amino acid aminotransferase
BBB	blood–brain barrier
BPF	Bisphenol F
CA	cinnabarinic acid
CKD	chronic kidney disease
CNS	central nervous system
CrPic	chromium picolinate
GPR35	G-protein-coupled receptor 35
3-HAA	3-hydroxyanthranilic acid
HD	Huntington’s disease
3-HK	3-hydroxykynurenine
IAA	indole-3-acetic acid
IaaH	indole-3-acetamide hydrolase
IAAld	indole-3-acetaldehyde
IAcA	3-indole acrylic acid
IAld	indole-3-aldehyde
IAM	indole-3-acetamide
IDO	indoleamine 2,3-dioxygenase
ILA	indole-3-lactic acid
INS	indoxyl sulfate
IPA	indole-3-propionic acid
IPyA	indole-3-pyruvic acid
JAK/STAT	Janus kinase/signal transducers and activators of transcription
KATs	kynurenine aminotransferases
KYN	kynurenine
KYNA	kynurenic acid
LPS	lipopolysaccharide
MAPK	mitogen-activated protein kinase
mGluRs	metabotropic glutamate receptors
MS	multiple sclerosis
NAD	nicotinamide adenine dinucleotide
NMDA	N-methyl-D-aspartate
PA	picolinic acid
PD	Parkinson’s disease
QUIN	quinolinic acid
ROS	reactive oxygen species
TDO	tryptophan 2,3-dioxygenase
TEAC	Trolox equivalent antioxidant capacity
TMO	tryptophan-2-monooxygenase
TNA	tryptophanase
TrD	tryptophan decarboxylase
Tregs	regulatory T cells
Trp	tryptophan
XA	xanthurenic acid
